# The identification of high-preforming antibodies for Ubiquilin-2 for use in Western Blot, immunoprecipitation, and immunofluorescence

**DOI:** 10.12688/f1000research.131851.1

**Published:** 2023-03-30

**Authors:** Ian McDowell, Riham Ayoubi, Maryam Fotouhi, Kathleen Southern, Peter S. McPherson, Carl Laflamme, NeuroSGC/YCharOS/EDDU collaborative group, ABIF Consortium

**Affiliations:** 1 Department of Neurology and Neurosurgery, Structural Genomics Consortium, The Montreal Neurological Institute, McGill University, Montreal, Quebec, H3A 2B4, Canada

**Keywords:** Uniprot ID Q9UHD9, UBQLN2, Ubiquilin-2, antibody characterization, antibody validation, Western Blot, immunoprecipitation, immunofluorescence

## Abstract

Ubiquilin-2, a member of the ubiquilin protein family, plays a role in the regulation of various protein degradation pathways, and is mutated in some neurodegenerative diseases. Well-characterized anti-Ubiquilin-2 antibodies would advance reproducible research for Ubiquilin-2 and in turn, benefit the scientific community. In this study, we characterized ten Ubiquilin-2 commercial antibodies for Western Blot, immunoprecipitation, and immunofluorescence using a standardized experimental protocol based on comparing read-outs in knockout cell lines and isogenic parental controls. We identified many high-performing antibodies and encourage readers to use this report as a guide to select the most appropriate antibody for their specific needs.

## Introduction

Ubiquilin-2, a protein encoded by the *UBQLN2* gene, plays a critical role in protein degradation pathways; including the ubiquitin-proteasome system (UPS), autophagy and the endoplasmic reticulum-associated protein degradation (ERAD) pathway. ^1^

Disease-causing variants of *UBQLN2* have been identified in patients suffering from amyotrophic lateral sclerosis and frontotemporal dementia (ALS/FTD). ^2^ These *UBQLN2* mutations are predicted to be acting on ALS pathological mechanisms by causing UPS and autophagy dysfunction, ^3^ neuroinflammation ^4^^,^^5^ and/or formation of stress granules. ^6^^,^^7^ Mechanistic studies would be greatly facilitated with the availability of high-quality antibodies.

Here, we compared the performance of a range of commercially available antibodies for Ubiquilin-2 and validated high-quality antibodies for Western Blot, immunoprecipitation and immunofluorescence, enabling biochemical and cellular assessment of Ubiquilin-2 properties and function.

## Results and discussion

Our standard protocol involved comparing readouts from wild-type (WT) and knockout (KO) cells. ^8^^,^^9^ The first step was to identify a cell line(s) that expresses sufficient levels of Ubiquilin-2 to generate a measurable signal. To this end, we examined the DepMap transcriptomics database to identify all cell lines that express *UBQLN2* at levels greater than 2.5 log _2_ (transcripts per million “TPM” +1), which we had found to be a suitable cut-off (Cancer Dependency Map Portal, RRID:SCR_017655). Commercially available HAP1 cells expressed the *UBQLN2* transcript at RNA levels above the average range of cancer cells analyzed. The parental and *UBQLN2* KO HAP1 cells were obtained from Horizon Discovery ( Table 1).

**Table 1.** Summary of the cell lines used.

Institution	Catalog number	RRID (Cellosaurus)	Cell line	Genotype
Horizon Discovery	C631	CVCL_Y019	HAP1	WT
Horizon Discovery	HZGHC004089c001	CVCL_TW09	HAP1	*UBQLN2* KO

**Figure 1.**
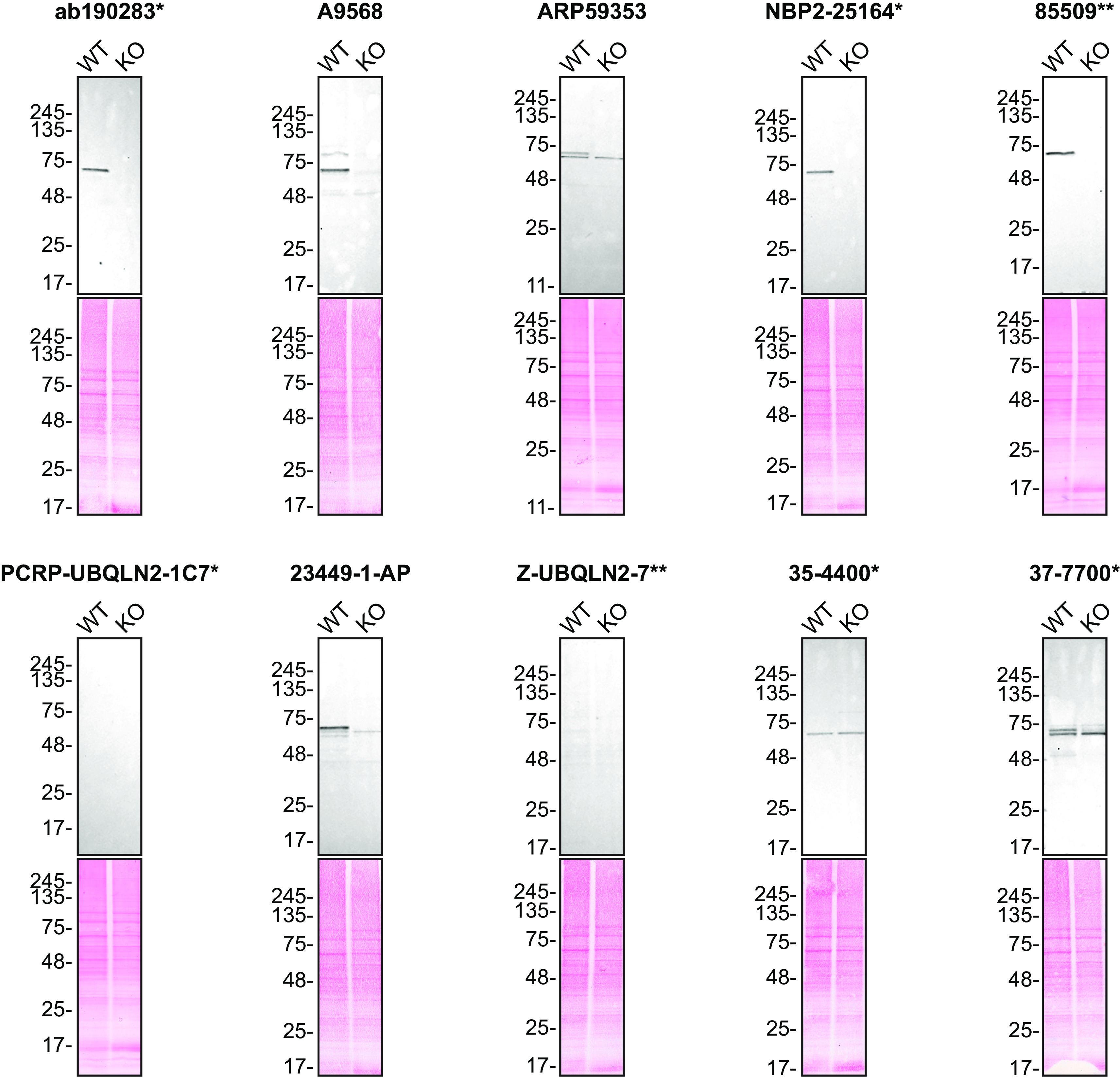
Ubiquilin-2 antibody screening by Western Blot. Lysates of HAP1 wild-type (WT) and *UBQLN2* knockout (KO) were prepared, and 30 μg of protein were processed for Western Blot with the indicated Ubiquilin-2 antibodies. The Ponceau stained transfers of each blot are presented to show equal loading of WT and KO lysates and protein transfer efficiency from the acrylamide gels to the nitrocellulose membrane. Antibody dilutions were chosen according to the recommendations of the antibody supplier. When the concentration was not indicated by the supplier, which was the case for antibody 35-4400*, we tested it at 1/1000. Antibody dilution used: ab190283* at 1/2000, A9568 at 1/1000, ARP59353 at 1/500, NBP2-25164* at 1/2000, 85509** at 1/1000, PCRP-UBQLN2-1C7* at 1/26, 23449-1-AP at 1/1000, Z-UBQLN2-7** at 1/653, 35-4400* at 1/1000, and 37-7700* at 1/2000. Predicted band size: 65 kDa. *Monoclonal antibody, **Recombinant antibody.

For Western Blot experiments, we resolved proteins from WT and *UBQLN2* KO cell extracts and probed them side-by-side with all antibodies in parallel ( Figure 1).

**Figure 2.**
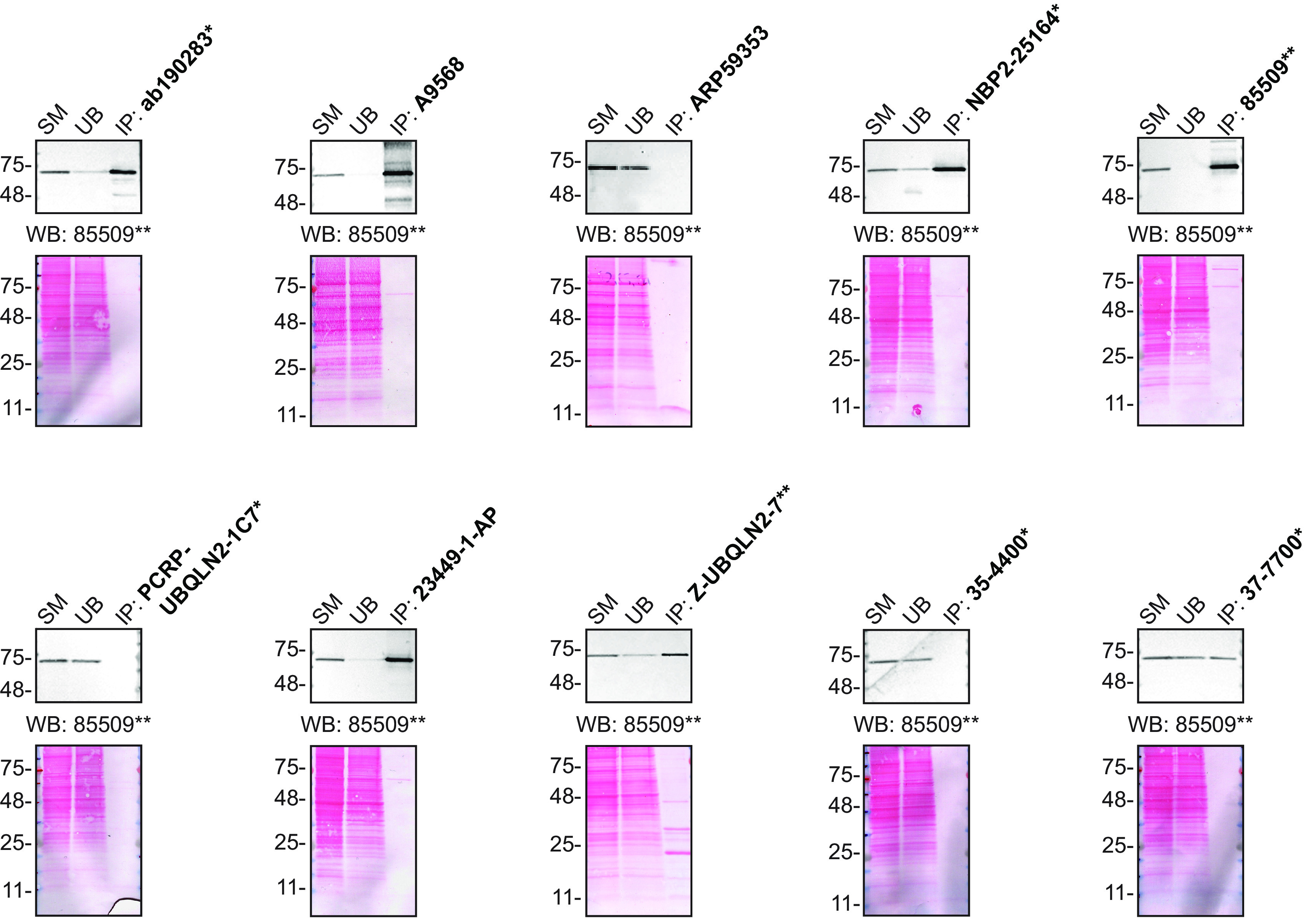
Ubiquilin-2 antibody screening by immunoprecipitation. HAP1 lysates were prepared, and IP was performed using 2.0 μg of the indicated Ubiquilin-2 antibodies pre-coupled to Dynabeads protein G or protein A or Flag-M2 magnetic beads. Samples were washed and processed for Western Blot with the indicated Ubiquilin-2 antibody. For Western Blot, 85509** was used at 1/1000. The Ponceau stained transfers of each blot are shown for similar reasons as in Figure 1. SM=4% starting material; UB=4% unbound fraction; IP=immunoprecipitate. *Monoclonal antibody, **Recombinant antibody.

For immunoprecipitation experiments, we used each of the antibodies to immunopurify Ubiquilin-2 from HAP1 cell extracts. The performance of each antibody was evaluated by detecting the Ubiquilin-2 protein in extracts, in the immunodepleted extracts and in the immunoprecipitates ( Figure 2).

**Figure 3.**
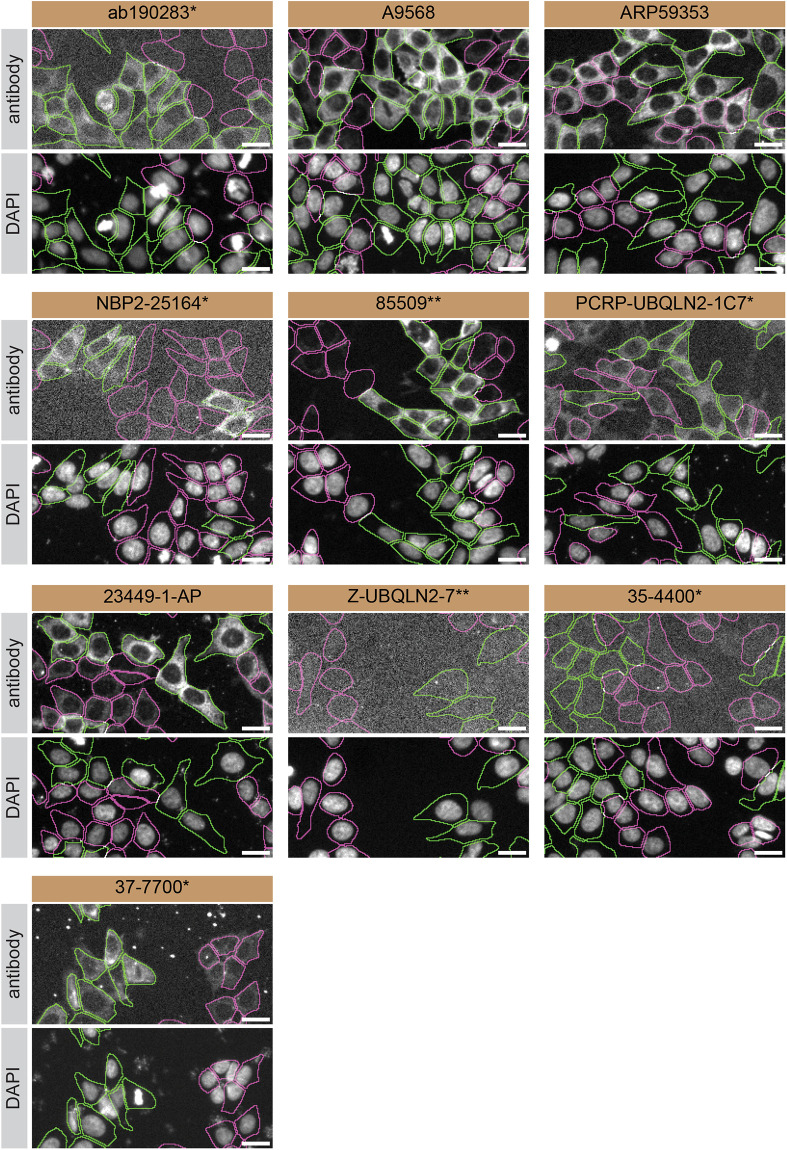
Ubiquilin-2 antibody screening by immunofluorescence. HAP1 wild-type (WT) and *UBQLN2* knockout (KO) cells were labelled with a green or a far-red fluorescent dye, respectively. WT and KO cells were mixed and plated to a 1:1 ratio in a 96-well plate with glass bottom. Cells were stained with the indicated Ubiquilin-2 antibodies and with the corresponding Alexa-fluor 555 coupled secondary antibody including DAPI. Acquisition of the blue (nucleus-DAPI), green (identification of WT cells), red (antibody staining) and far-red (identification of KO cells) channels was performed. Representative images of the merged blue and red (grayscale) channels are shown. WT and KO cells are outlined with green and magenta dashed line, respectively. When the concentration was not indicated by the supplier, which was the case for antibodies ARP59353, 85509**, PCRP-UBQLN2-1C7* and Z-UBQLN2-7**, we tested them at 1/500, 1/200, 1/20 and 1/600, respectively. At these concentrations, the signal from each antibody was in the range of detection of the microscope used. Antibody dilution used: ab190283* at 1/1000, A9568 at 1/2000, ARP59353 at 1/500, NBP2-25164* at 1/1000, 85509** at 1/200, PCRP-UBQLN2-1C7* at 1/20, 23449-1-AP at 1/300, Z-UBQLN2-7** at 1/600, 35-4400* at 1/500, and 37-7700* at 1/500. Bars = 10 μm. *Monoclonal antibody, **Recombinant antibody.

For immunofluorescence experiments, as described previously, antibodies were screened using a mosaic strategy. ^10^ In brief, we plated WT and KO cells together in the same well and imaged both cell types in the same field of view to reduce staining, imaging and image analysis biases ( Figure 3).

In conclusion, we have screened Ubiquilin-2 commercial antibodies by Western Blot, immunoprecipitation and immunofluorescence and identified several high-performing antibodies under our standardized experimental conditions. The underlying data is uploaded to Zenodo. ^16^^,^^17^

## Methods

### Antibodies

All Ubiquilin-2 antibodies are listed in Table 2, together with their corresponding Research Resource Identifiers (RRID), to ensure the antibodies are cited properly. ^11^ Peroxidase-conjugated goat anti-mouse and anti-rabbit antibodies are from Thermo Fisher Scientific (cat. number 62-6520 and 65-6120). Peroxidase-conjugated monoclonal anti-Flag M2 is from MilliporeSigma (cat. number A8592). Alexa-555-conjugated goat anti-mouse and anti-rabbit secondary antibodies are from Thermo Fisher Scientific (cat. number A21424 and A21429). The anti-FLAG (M2 clone) conjugated with Cy3 is from MilliporeSigma (cat. number A9594).

**Table 2.** Summary of the Ubiquilin-2 antibodies tested.

Company	Catalog number	Lot number	RRID (Antibody Registry/Addgene)	Clonality	Clone ID	Host	Concentration (μg/μl)	Vendors recommended applications
Abcam	ab190283 *	GR3297673-4	AB_2747782	monoclonal	6H9	mouse	1.00	Wb, IF
ABclonal	A9568	92790201	AB_2772790	polyclonal	-	rabbit	2.42	Wb, IF
Aviva Systems Biology	ARP59353	QC30343-40589	AB_10865653	polyclonal	-	rabbit	0.50	Wb
Bio-techne	NBP2-25164 *	81919	AB_2885154	monoclonal	6H9	mouse	1.00	Wb, IF
Cell Signaling Technology	85509 **	1	AB_2800056	recombinant-mono	D7R2Z	rabbit	0.12	Wb, IP
Developmental Studies Hybridoma Bank	PCRP-UBQLN2-1C7 *	2021-03-04	AB_2619216	monoclonal	PCRP-UBQLN2-1C7	mouse	0.03	Wb, IP
Proteintech	23449-1-AP	52217	AB_2879282	polyclonal	-	rabbit	0.55	Wb, IF
Structural Genomics Consortium	Z-UBQLN2-7 **	YSUBQLN2A-c001	Addgene_166563	recombinant-mono	YSUBQLN2A-c001	human	0.65	IP
Thermo Fisher Scientific	35-4400 *	WA320043	AB_2533204	monoclonal	3B8A10	mouse	0.50	Wb, IF
Thermo Fisher Scientific	37-7700 *	VA300207	AB_2533341	monoclonal	3D5E2	mouse	0.50	Wb, IP, IF

Wb=Western blot; IF=immunofluorescence; IP=immunoprecipitation.

* Monoclonal antibody.

** Recombinant antibody.

### Cell culture

Both HAP1 WT and *UBQLN2* KO cell lines used are listed in Table 1, together with their corresponding RRID, to ensure the cell lines are cited properly. ^12^ Cells were cultured in DMEM high glucose (GE Healthcare cat. number SH30081.01) containing 10% fetal bovine serum (Wisent, cat. number 080450), 2 mM L-glutamate (Wisent cat. number 609065), 100 IU penicillin and 100 μg/ml streptomycin (Wisent cat. number 450201).

### Antibody screening by Western Blot

Western Blot experiments were performed as described in our standard operating procedure. ^13^ HAP1 WT and *UBQLN2* KO were collected in RIPA buffer (25 mM Tris-HCl pH 7.6, 150 mM NaCl, 1% NP-40, 1% sodium deoxycholate, 0.1% SDS) (Thermo Fisher Scientific, cat. number 0089901), supplemented with 1× protease inhibitor cocktail mix (MilliporeSigma, cat. number 78429). Lysates were sonicated briefly and incubated for 30 min on ice. Lysates were spun at ~110,000× g for 15 min at 4°C and equal protein aliquots of the supernatants were analyzed by SDS-PAGE and immunoblot. BLUelf prestained protein ladder from GeneDireX (cat. number PM008-0500) was used.

Western Blots were performed with precast midi 4-20% Tris-Glycine polyacrylamide gels from Thermo Fisher Scientific (cat. number WXP42012BOX) ran with Tris/Glycine/SDS buffer from bio-Rad (cat. number 1610772), loaded in Laemmli loading sample buffer from Thermo Fisher Scientific (cat. number AAJ61337AD) and transferred on nitrocellulose membranes. Proteins on the blots were visualized with Ponceau staining which is scanned to show together with individual Western Blots. Blots were blocked with 5% milk for 1 hr, and antibodies were incubated overnight at 4°C with 5% bovine serum albumin (BSA) (Wisent, cat. number 800-095) in TBS with 0.1% Tween 20 (TBST) (Cell Signalling Technology, cat. number 9997). Following three washes with TBST, the peroxidase conjugated secondary antibody was incubated at a dilution of ~0.2 μg/ml in TBST with 5% milk for 1 hr at room temperature followed by three washes with TBST. Membranes were incubated with Pierce ECL (Thermo Fisher Scientific, cat. number 32106) prior to detection with the iBright™ CL1500 Imaging System (Thermo Fisher Scientific, cat. number A44240).

### Antibody screening by immunoprecipitation

Immunoprecipitation experiments were performed as described in our standard operating procedure. ^14^ Antibody-bead conjugates were prepared by adding 2 μg to 500 μl of Pierce IP Lysis Buffer from Thermo Fisher Scientific (cat. number 87788) in a 1.5 mL microcentrifuge tube, together with 30μl of Dynabeads protein A- (for rabbit antibodies) or protein G- (for mouse antibodies) from Thermo Fisher Scientific (cat. number 10002D and 10004D, respectively) or anti-Flag M2 magnetic beads from MilliporeSigma (cat. number M8823). Tubes were rocked for ~1 hr at 4°C followed by two washes to remove unbound antibodies.

HAP1 WT were collected in Pierce IP buffer (25 mM Tris-HCl pH 7.4, 150 mM NaCl, 1 mM EDTA, 1% NP-40 and 5% glycerol) (Thermo Fisher Scientific, cat. number 87788) supplemented with protease inhibitor (Millipore Sigma, cat. number P8340). Lysates were rocked 30 min at 4°C and spun at 110,000× g for 15 min at 4°C. 0.5 ml aliquots at 2.0 mg/ml of lysate were incubated with an antibody-bead conjugate for ~1 hr at 4°C. The unbound fractions were collected, and beads were subsequently washed three times with 1.0 ml of IP buffer and processed for SDS-PAGE and Western Blot on precast midi 4-20% Tris-Glycine polyacrylamide gels (Thermo Fisher Scientific, cat number WXP42012BOX).

### Antibody screening by immunofluorescence

Immunofluorescence was performed as described in our standard operating procedure. ^10^ HAP1 WT and *UBQLN2* KO were labelled with CellTracker ^TM^ green (Thermo Fisher Scientific, cat. number C2925) or CellTracker ^TM^ deep red (Thermo Fisher Scientific, cat. number C34565) fluorescence dye, respectively. The nuclei were labelled with DAPI (Thermo Fisher Scientific, cat. Number D3571) fluorescent stain. WT and KO cells were plated in 96 well glass plates (Perkin Elmer, cat. number 6055300) as a mosaic and incubated for 24 hrs in a cell culture incubator at 37 ^o^C, 5% CO _2_. Cells were fixed in 4% paraformaldehyde (PFA) (Beantown chemical, cat. number 140770-10ml) in phosphate buffered saline (PBS) (Wisent, cat. number 311-010-CL) for 15 min at room temperature and then washed 3 times with PBS. Cells were permeabilized in PBS with 0.1% Triton X-100 (Thermo Fisher Scientific, cat. number BP151-500) for 10 min at room temperature and blocked with PBS containing 5% BSA, 5% goat serum (Gibco, cat. number 16210-064) and 0.01% Triton X-100 for 30 min at room temperature. Cells were incubated with IF buffer (PBS, 5% BSA, 0.01% Triton X-100) containing the primary Ubiquilin-2 antibodies overnight at 4°C. Cells were then washed 3 × 10 min with IF buffer and incubated with the corresponding Alexa Fluor 555-conjugated secondary antibodies in IF buffer at a dilution of 1.0 μg/ml for 1 hr at room temperature with DAPI. Cells were washed 3 × 10 min with IF buffer and once with PBS.

Images were acquired on an ImageXpress micro widefield high-content microscopy system (Molecular Devices), using a 20×/0.45 NA air objective lens and scientific CMOS camera (16-bit, 1.97mm field of view), equipped with 395, 475, 555 and 635 nm solid state LED lights (Lumencor Aura III light engine) and bandpass emission filters (432/36 nm, 520/35 nm, 600/37 nm and 692/40 nm) to excite and capture fluorescence emission for DAPI, CellTracker ^TM^ Green, Alexa fluor 555 and CellTracker ^TM^ Red, respectively. Images had pixel sizes of 0.68 × 0.68 microns. Exposure time was set with maximal (relevant) pixel intensity ~80% of dynamic range and verified on multiple wells before acquisition. Since the IF staining varied depending on the primary antibody used, the exposure time was set using the most intensely stained well as reference. Frequently, the focal plane varied slightly within a single field of view. To remedy this issue, a stack of three images per channel was acquired at a z-interval of 4 microns per field and best focus projections were generated during the acquisition (MetaExpress v6.7.1, Molecular Devices). Segmentation was carried out on the projections of CellTracker ^TM^ channels using CellPose v1.0 on green (WT) and far-red (KO) channels, using as parameters the ‘cyto’ model to detect whole cells, and using an estimated diameter tested for each cell type, between 15 and 20 microns. ^15^ Masks were used to generate cell outlines for intensity quantificationFigures were assembled with Adobe Illustrator.

## Data Availability

Zenodo: Antibody Characterization Report for Ubiquilin-2, https://doi.org/10.5281/zenodo.7459541. ^16^ Zenodo: Dataset for the Ubiquilin-2 antibody screening study, https://doi.org/10.5281/zenodo.7671135. ^17^ Data are available under the terms of the Creative Commons Attribution 4.0 International license (CC-BY 4.0).
