# Supplementary material for: The identification of high-preforming antibodies for Ubiquilin-2 for use in Western Blot, immunoprecipitation, and immunofluorescence
Source: F1000Res. 2023 Mar 30;12:355. [Version 1] doi: 10.12688/f1000research.131851.1 (PMC10285353; doi:10.12688/f1000research.131851.1)
Supplement: Peer review history

# Peer review: Reviewer response for version 1

**DOI:** 10.5256/f1000research.144734.r173280

**Published:** 2023-06-08

**Authors:** Carlos Castaneda

**Affiliations:** 1 Departments of Biology and Chemistry, Syracuse University, Syracuse, New York, USA

This data note is a very important resource for the ubiquilin (UBQLN) community, as it does a very good job systematically comparing ten commercially available UBQLN2 antibodies. UBQLN2 is an important disease-linked protein whose functions are connected with the ubiquitin-proteasome system, autophagy, and stress-induced condensates. The study raises the important awareness that there are substantial differences in how these antibodies detect UBQLN2 in cell lysates, immunoprecipitation assays, and immunofluorescence experiments that examine subcellular localization of UBQLN2. The use of standardized controls and procedures enables a clear delineation as to which antibodies work best and which do not. It will be an excellent reference for many labs investigating UBQLN2 function in cells. There are only a couple of suggestions to further improve this note:

1. Include information (if available) on antibody epitope region; this will help ascertain whether antibodies potentially detect other related UBQLN paralogs (e.g. UBQLN1 or UBQLN4).
2. In the introduction, rephrase “and /or regulation of stress granules”.
3. The title should be reworded to say ‘high-performing’ (performing is misspelled).

Are sufficient details of methods and materials provided to allow replication by others?

Yes

Is the rationale for creating the dataset(s) clearly described?

Yes

Are the datasets clearly presented in a useable and accessible format?

Yes

Are the protocols appropriate and is the work technically sound?

Yes

Reviewer Expertise:

biophysics, protein structure and function, biomolecular condensates, protein quality control, and ubiquitin biology.

I confirm that I have read this submission and believe that I have an appropriate level of expertise to confirm that it is of an acceptable scientific standard.

# Peer review: Reviewer response for version 1

**DOI:** 10.5256/f1000research.144734.r173282

**Published:** 2023-05-26

**Authors:** Mervyn J. Monteiro

**Affiliations:** 1 Center for Biomedical Engineering and Technology, University of Maryland School of Medicine, Baltimore, MD, USA

This is an excellent study comparing the utility of different commercial UBQLN2 antibodies. The authors have tested the utility and specificity of these antibodies for Western blotting, immunoprecipitation and immunofluorescence staining using a UBQLN2 expressing and knockout cell lines. Readers will be able to determine which of the antibodies are specific for UBQLN2. One limitation of the approach is the choice of the cell line, which seems to express little UBQLN4. The lack of its expression makes it difficult to assess if the UBQLN2 antibodies cross-react with UBQLN4.

Are sufficient details of methods and materials provided to allow replication by others?

Yes

Is the rationale for creating the dataset(s) clearly described?

Yes

Are the datasets clearly presented in a useable and accessible format?

Yes

Are the protocols appropriate and is the work technically sound?

Yes

Reviewer Expertise:

Neurodegenerative disorders

I confirm that I have read this submission and believe that I have an appropriate level of expertise to confirm that it is of an acceptable scientific standard.
